# Development and Characterization of Electrospun Poly(3-hydroxybutyrate-co-3-hydroxyhexanoate) (PHBH) Biopapers

**DOI:** 10.3390/polym18091061

**Published:** 2026-04-28

**Authors:** Ahmet Ozan Basar, Cristina Prieto, Luis Cabedo, Chris Sammon, Jose Maria Lagaron

**Affiliations:** 1 Novel Materials and Nanotechnology Group, Institute of Agrochemistry and Food Technology (IATA), Spanish Council for Scientific Research (CSIC), 46980 Paterna, Spain; cprieto@iata.csic.es; 2 PhD in Food Science, Technology and Management, Universitat Politècnica de València, 46022 Valencia, Spain; 3 Polymers and Advanced Materials Group (PIMA), School of Technology and Experimental Sciences, Universitat Jaume I (UJI), 12071 Castellón, Spain; lcabedo@uji.es; 4 Materials and Engineering Research Institute, Sheffield Hallam University, Sheffield S1 1WB, UK; c.sammon@shu.ac.uk

**Keywords:** PHBH, electrospinning, biopapers, compression molding, food packaging

## Abstract

In this study, electrospun poly(3-hydroxybutyrate-co-3-hydroxyhexanoate) (PHBH) biopapers were produced by annealing electrospun fiber mats from two commercial grades (151C and X131A) and compared with films prepared by the conventional melt-mixing/compression molding method. To obtain continuous biopapers, the fiber mats were subjected to mild thermal post-processing at various temperatures. The selected annealing temperatures were 140 °C (151C) and 130 °C (X131A), where interfiber coalescence occurred within a short annealing time (10 s), yielding continuous fibrous films (biopapers). To elucidate the structural mechanisms underlying interfiber coalescence, time-resolved synchrotron SAXS/WAXS and temperature-dependent FTIR spectroscopy were performed. These analyses showed that coalescence occurred through an interplay between thermally induced local ordering at sub-melting temperatures and premelting/partial melting of thin, ill-defined lamellae, with grade-dependent contributions. The resulting biopapers were evaluated against compression-molded films for optical, mechanical, and barrier properties relevant to packaging. All samples showed similar transparency, although compression-molded films were slightly more opaque. The lower-rigidity grade (151C) exhibited more ductile and tougher behavior than X131A. Biopapers showed slightly lower water and oxygen barrier performance than compression-molded films, attributed to differences in material compactness. Overall, brief mild annealing after electrospinning enabled continuous PHBH biopapers with balanced properties, supporting their potential for sustainable PHBH-based food-packaging applications.

## 1. Introduction

Polymeric materials play a fundamental role in numerous industrial sectors, ranging from packaging to biomedical [1,2]. However, the widespread use of single-use plastics has resulted in significant environmental concerns, prompting the need for stricter regulations and innovative solutions [3,4]. In response to this global issue, the European Union has implemented stringent regulations to curb the use of single-use plastics and promote sustainable practices [5]. These regulations are designed to mitigate plastic waste and encourage the adoption of the Circular Economy based on the “reuse, reduce, and recycle” principle. However, the “recycle” step is currently being reconsidered, with a focus on shifting towards sustainable alternatives to petroleum-based polymers due to the low demand and efficiency of recycled plastics [6]. In light of this, significant research efforts have been directed towards the development of sustainable biodegradable polymers derived from renewable resources, aligning more closely with the Circular Economy concept. Among these alternatives, polyhydroxyalkanoates (PHAs) constitute a class of biobased, biocompatible, compostable, and biodegradable polymers [7,8,9]. These polymers are produced through microbial fermentation of renewable resources, including different types of organic waste streams [7,10]. This is particularly important in the sense of mitigating, at least partially, environmental concerns by reducing the reliance on fossil fuels and minimizing carbon emissions. Furthermore, the production of PHAs from renewable feedstocks ensures that it avoids competition with the food chain or with the use of land.

PHAs are generally linear aliphatic polyesters made up of repeating hydroxy acids (HAs) linked by ester bonds [11]. PHAs can be categorized based on the quantity of carbon atoms in their constituent monomers, with short chain length (scl) encompassing monomers containing 3–5 carbon atoms, and medium chain length (mcl) comprising those with 6–14 carbon atoms [11,12]. Typical examples of scl-PHAs are poly(3-hydroxybutyrate) (PHB), poly(3-hydroxybutyrate-co-3-hydroxyvalerate (PHBV), and poly(3-hydroxybutyrate-co-4-hydroxybutyrate) [P(3HB-co-4HB)], whereas the common mcl-PHAs include poly(3-hydroxybutyrate-co-3-hydroxyhexanoate) (PHBH) and poly(3-hydroxyhexanoate-co-3-hydroxyoctanoate) (PHHO) [11,12]. One of the most extensively studied and commercially available PHAs is the homopolymer poly(3-hydroxybutyrate) (PHB), which exhibits favorable thermal, mechanical, and biocompatible properties, along with excellent gas barrier characteristics [13,14]. However, its application in packaging is often hindered by various factors. Firstly, PHB faces challenges in terms of poor processability, as its degradation temperature (200 °C) is close to its melting point (~180 °C) [15]. Secondly, the high degree of crystallinity (50–70%) and macromolecular organization contribute to its rigidity and brittleness [16]. To address these limitations, a common approach entails synthesizing copolymers of PHB through the inclusion of comonomers belonging to the PHA family, such as hydroxyvalerate (3HV) and hydroxyhexanoate (3HH) units [9,17]. The scl-copolyester poly(3-hydroxybutyrate-co-3-hydroxyvalerate) (PHBV) is one of the most intensively studied PHA copolyesters and is readily available in the market with low 3HV contents (<12 wt.%) [18]. Depending on the 3HV content, PHBV demonstrates enhanced properties such as reduced crystallinity and brittleness, a wider processing window, and superior mechanical characteristics compared to PHB [9]. However, both PHB and low 3HV content PHBV polymers suffer from inherent fragility that worsens over time due to secondary crystallization and physical aging [19]. Furthermore, their high crystallinity imposes limitations on their processability in conventional industrial equipment, resulting in a narrow processing window [20].

Poly(3-hydroxybutyrate-co-3-hydroxyhexanoate) (PHBH) is of great interest due to its ability to tailor the thermo-mechanical properties by adjusting the content of highly crystalline units (3HB) and non-crystallizable mcl elastomeric units (3HH) in the copolymer [9,21]. The inclusion of propyl groups in PHBH disrupts the polymer’s regular structure, leading to a reduction in crystallinity and melting temperature as the content of 3HH units increases. This reduction in the melting point widens the processing window of PHBH, minimizing thermal degradation of the polymer during processing. Meanwhile, the lower crystallinity enables the material to possess a more ductile and tough nature, while still maintaining the sought-after properties of compostability and complete biodegradability [9,21]. However, PHBH has received limited attention compared to its homopolymer and copolymer counterparts, primarily attributed to factors such as its restricted availability in the market, high production costs, and ongoing advancements in the synthesis process [9].

In the field of PHAs, various methods are employed in the production of packaging materials, including casting, extrusion, thermoforming, injection molding, welding, foaming, and blow molding [22]. Nevertheless, traditional approaches have inherent limitations, such as the requirement for high temperatures, the production of thick layers, inconsistency in additive distribution, instability of active compounds during processing, and challenges in scaling up, etc. [23]. In this regard, electrospinning technology emerges as a pioneering technique for the fabrication of materials with significant relevance in food packaging applications. The electrospinning process involves the vaporization of organic solvents of a liquid polymeric solution under the influence of a high electrical potential, resulting in the formation of dry micro- or nanofibers at ambient temperature [24,25]. These fibrous mats can subsequently be converted into continuous films by means of a mild thermal post-processing step, known as annealing, carried out for a short time and below the melting point of the biopolymer. Consequently, this methodology circumvents the occurrence of thermal degradation in PHAs during the processing stage. Recently, Cherpinski et al. conducted a study demonstrating a novel approach for transforming electrospun PHB fiber mats into continuous films, termed biopapers, through annealing [26]. In a more recent follow-up study, Melendez-Rodriguez et al. also developed electrospun biopapers using the same annealing method but a different kind of PHA, particularly PHBV with varying 3HV contents [27]. A common point of these two disruptive studies is that the annealing process occurs below the melting point of the biopolymer for a brief period (5–10 s), expected to minimize any potential thermal degradation, and the resulting electrospun biopapers, which retain a distinctive fibrous locked morphology, are specifically designed for sustainable barrier applications, including food packaging [27,28]. Furthermore, from an industrial perspective, recent advances in electrospinning scale-up, including multi-needle and needleless systems, support the viability of this approach for high-throughput production [29]. In this context, the electrospinning-plus-annealing route is especially attractive when reduced thermal load is required, for instance for biopolymers with limited thermal stability and narrow processing windows, or for the incorporation of thermolabile functional ingredients that could be compromised during conventional melt processing. In addition, this approach enables thinner films while maintaining packaging-relevant properties in the same practical range as films produced by conventional melt-processing methods.

In this study, electrospun PHBH biopapers were developed through mild thermal post-treatment using two different commercial PHBH grades, 151C and X131A. The resulting materials were characterized in terms of morphology, crystallinity, temperature-dependent crystalline morphology, and other physicochemical properties relevant to food packaging applications. The electrospun biopapers were also compared with films produced from the same grades by melt-compounding followed by compression molding, in order to benchmark this mild route against a conventional melt-processing reference, provide fundamental insight into electrospun-to-film transformation in PHBH systems, and evaluate the practical relevance of this approach for packaging applications.

## 2. Materials and Methods

### 2.1. Materials

Two different grades of commercial poly(3-hydroxybutyrate-co-3-hydroxyhexanoate) (PHBH), as commercial names of Aonilex 151C and Aonilex X131A, were purchased from Kaneka (Osaka, Japan) and delivered in the form of pellets. According to the manufacturer, the density of the polymers was 1.19 g/cm^3^ and 1.20 g/cm^3^ for 151C and X131A, respectively. The (R)-3-hydroxyhexanoate (3HH) content was previously reported as 11 mol.% and 6 mol.% for 151C and X131A, respectively [14]. 2,2,2-trifluorethanol (TFE) (≥99% purity) was purchased from Merck (Darmstadt, Germany).

### 2.2. Solution Preparation and Characterization

For each grade of PHBH (151C and X131A), electrospinning solutions were prepared by dissolving 10 wt.% of polymer in TFE at 50 °C through gentle stirring overnight. Prior to electrospinning, the physical properties of the polymeric solutions were characterized in terms of viscosity, surface tension, and conductivity. The viscosity was measured using an IKA Rotavisc lo-vi viscosity meter (Staufen, Germany). Surface tension measurements were carried out using the Wilhelmy plate method in the Krüss GmbH EasyDyne K20 tensiometer (Hamburg, Germany). Finally, the conductivity was measured using a Hanna Instruments HI98192 conductivity probe (Gothenburg, Sweden). All measurements were done in triplicate at room temperature.

### 2.3. Electrospinning and Annealing Processes

All electrospinning processes were performed using an electrospinning apparatus, high-throughput Fluidnatek LE-500 pilot line from Bioinicia S.L. (Valencia, Spain), which is equipped with a scanning single needle injector. For the electrospinning of two different grades of PHBH, all parameters were the same. Each solution was placed into the electrospinning equipment within a 20 mL plastic syringe coupled by a PTFE tube to a stainless-steel needle (internal diameter of 0.4 mm), which was connected to the voltage supply. The used electrospinning parameters were: 23 cm of distance between the needle and the collector, 6 mL/h of flow rate, and +10 kV of voltage in the injector and −3 kV of voltage in the collector, for each solution. Both solutions were electrospinable and stable under these processing conditions. All PHBH solutions were electrospun for 2 h to achieve a desired thickness of the fiber mats at room temperature and 40% relative humidity (RH) conditions. The produced mats were then preserved in a desiccator at room temperature and at 0% RH for at least two weeks prior to the annealing process.

For the annealing method, obtained fiber mats were subjected to a thermal post-treatment using a 4122-model press from Carver, Inc. (Wabash, IN, USA). The annealing was performed between 100 °C and 170 °C, for 10 s and without applying pressure, in order to identify, for each grade, the lowest temperature that yielded a continuous biopaper with very low residual porosity by SEM. An average thickness of 80 μm was yielded for each sample.

### 2.4. Preparation of Films by Compression Molding

For the case of preparation of compression-molded PHBH films, prior to processing, both grades of PHBH pellets were dried at 60 °C for 24 h to eliminate any possible residual moisture, using a vacuum oven (Digiheat, JP Selecta S.A., Barcelona, Spain). Then, the dried pellets, a total amount of 10 g each, were processed in a 16 cm^3^ melt-mixer, specifically the Plastograph Original E Internal Mixer from Brabender GmbH & Co. KG (Duisburg, Germany), and it was set to a rotating speed of 60 rpm for 3 min. The processing temperature was 175 °C and 155 °C for 151C and X131A grades, respectively. After the completion of the mixing process, each batch was extracted from the mini-mixer and subjected to cooling at room temperature. For the conditioning process, the resulting doughs were placed in a desiccator containing silica gel at 0% RH and 25 °C for a minimum of 48 h.

The doughs of 151C and X131A PHBH were then thermo-compressed into films using the same hydraulic press from Carver (4122, Wabash, IN, USA). To ensure the thermal softening, the doughs of 151C grade were initially placed on the hot-plates at a temperature of 175 °C for 1 min, without applying any pressure. Afterward, the softened doughs were hot-pressed at a pressure of 3 tons for 2 min, followed by an increase in pressure to 6 tons for 1 min. For the X131A grade, the same procedure was followed, but using a temperature of 155 °C instead of 175 °C. For each PHBH grade, square flat films measuring 10 cm × 10 cm with an approximate thickness of 120 μm were obtained.

### 2.5. Characterizations

Unless otherwise stated, all fiber, biopaper, and compression-molded film samples were conditioned in a desiccator at 0% RH and 25 °C for 15 days prior to characterization.

#### 2.5.1. Morphology

The morphology of the electrospun fibers, biopapers, and compression-molded films was examined by scanning electron microscopy (SEM). For this, a Hitachi S-4800 electron microscope (Tokyo, Japan) was utilized at an accelerating voltage of 10 kV and a working distance of 8 mm. Prior to observations, the samples were put onto holders using conductive double-sided carbon tape and covered with a mixture of gold-palladium for 2 min under vacuum. The average fiber diameters were determined using ImageJ software (version 1.49, NIH, Bethesda, MD, USA) from a minimum of 10 SEM micrographs at their original magnifications.

For the cross-section imaging, the samples were cryo-fractured after immersion in liquid nitrogen and observed in SEM following the same methodology.

#### 2.5.2. Thermal Analysis

Thermogravimetric analysis (TGA) was performed for the fiber samples using a 550-TA Instruments thermogravimetric analyzer (New Castle, DE, USA). The program was set to heat from 25 to 700 °C with a rate of 10 °C/min under a nitrogen atmosphere. All thermogravimetric analyses were carried out using TRIOS software (version 4.4.0.41651, TA Instruments, New Castle, DE, USA).

Next, the main thermal parameters were determined for the PHBH-based electrospun fiber, biopapers, and compression-molded film samples using a Q20 differential scanning calorimetry (DSC) equipment from TA Instruments (New Castle, DE, USA). For this, approximately 3 mg of each sample was placed and sealed into a Tzero hermetic aluminum pan. Thermal runs consisted of a first heating step from −20 to 190 °C, a cooling step to −20 °C, and a second heating step to 190 °C. The runs were performed with a 10 °C/min heating rate under a nitrogen atmosphere. The DSC equipment was previously calibrated using indium as a standard, and an empty pan was used as a reference. All thermographs were analyzed using Universal Analysis 2000 software (version 4.5A, TA Instruments, New Castle, DE, USA). All thermal analyses, DSC and TGA, were performed in triplicate.

#### 2.5.3. ATR-FTIR Spectroscopy

Variable-temperature Fourier transform infrared (FTIR) spectroscopy analysis was carried out using a Nicolet Nexus FTIR instrument (Thermo Fisher Scientific, Wilmington, DE, USA) coupled with a variable-temperature single reflection diamond attenuated total reflectance (ATR) sampling accessory (Specac Ltd., Orpington, UK). Spectra were gathered by averaging 64 scans at a 0.5 cm^−1^ resolution, and the blank ATR crystal was used as background at the same temperature. Several factors, such as path length and molar extinction coefficient of the analyte, affect the intensity of the infrared spectrum. In the case of ATR geometry, as long as the sample-to-ATR crystal contact is consistent, the path length can be considered constant. In order to ensure that any alterations in peak intensity in the data were indicative of changes in the morphology of the samples, a calibrated torque wrench (Specac Ltd., Orpington, UK) was set to 80 cNm and used to clamp the samples directly onto the ATR crystal, delivering a load of approximately 350 N via the sample accessory anvil. Prior to measurements, the reproducibility values of both the sample contact and the resulting spectra intensity were validated. All spectra were obtained at 10 °C intervals from 30 to 60 °C, and at 5 °C intervals until 190 °C. To validate the chosen temperature, spectra acquisition was delayed until the digital reading on the temperature controller had completely stabilized.

#### 2.5.4. Time-Resolved Synchrotron Experiments

Simultaneous small-angle X-ray scattering (SAXS) and wide-angle X-ray scattering (WAXS) experiments were conducted for electrospun 151C and X131A PHBH fibers to investigate the temperature dependence. These experiments were performed at beamline BL11-noncrystalline diffraction (NCD) (WAXS/SAXS station) located at the ALBA synchrotron facilities in Barcelona, Spain. The calibration of SAXS and WAXS q-axes was achieved by measuring silver behenate (AgC_22_H_43_O_2_) and chromium (III) oxide (Cr_2_O_3_) standards, respectively. For data collection, two detectors were employed simultaneously: a photon counting detector Pilatus 1M from Dectris AG (Baden, Switzerland) for SAXS and a CDD WAXS LX255-HS detector from Rayonix, L.L.C. (Evanston, IL, USA) for WAXS. The incident wave had a wavelength (λ) of 1 Å. The distances between the sample and the SAXS and WAXS detectors were set at 6.6930 m and 0.1003 m, respectively, enabling a q-range of 0.021 to 2.240 nm^−1^ for SAXS and 3.67 to 92.19 nm^−1^ for WAXS. The photon flux delivered onto the sample at the beamline was greater than 1.5 × 1012 ph·s^−1^ at 12.4 keV, with a beam current of 150 mA. The beamline had a bandpass (ΔE/E) of 2.7 × 10^−4^ at 10.0 keV, and the beam size at the sample position was 349 μm × 379 μm.

In the in situ thermal experiments, electrospun fiber mats with a thickness of 100 μm were placed on a THMS600 hot stage from Linkam Scientific Instruments Ltd. (Epsom, UK). The samples underwent thermal ramps from 30 °C to 190 °C at a rate of 10 °C/min to analyze their evolution under thermal conditions.

#### 2.5.5. Transparency

The light transmission of the biopapers and compression-molded films was measured using 50 mm x 30 mm specimens in an ultraviolet-visible (UV-vis) spectrophotometer VIS3000 (Dinko Instruments, Barcelona, Spain). The quantification of light absorption was carried out at wavelengths between 200 and 700 nm. Transparency (*T*) and opacity (*O*) values were determined using Equations (1) [30] and (2) [31], respectively.

(1) $$T=\frac{A_{600}}{L}$$

(2) $$O=A_{500}.L$$

where *A*_600_ and *A*_500_ represent the absorbance values at 600 nm and 500 nm, respectively, and *L* corresponds to the sample thickness (mm). All measurements were performed in triplicate.

#### 2.5.6. WAXS

Wide-angle X-ray scattering (WAXS) analysis was conducted at room temperature for the PHBH-based biopapers and compression-molded films using a Bruker AXS D4 ENDEAVOR diffractometer (Billerica, MA, USA). The samples were examined in the reflection mode, employing incident Cu K-alpha radiation (k = 1.54 Å). The generator settings were adjusted to 40 kV and 40 mA. The data were collected over a scattering angle (2θ) range of 2–40°. The diffraction peaks were deconvoluted using Fityk software (version 1.3.1) [32]. The interplanar distances were calculated from the positions of the deconvoluted peaks using Bragg’s law, and the unit-cell parameters were estimated from the characteristic reflections. The degree of crystallinity was estimated from the ratio between the integrated area of the crystalline peaks and the total deconvoluted diffractogram area. Values are reported as estimated structural parameters from single measurements.

#### 2.5.7. Tensile Tests

Mechanical properties of the fiber, biopapers, and compression-molded film samples were characterized using a 4400 device from Instron (Norwood, MA, USA). Dumbbell-shaped specimens with a gauge section of 5 × 25 mm were tested at room conditions (25 °C and 50% RH) using a crosshead speed of 10 mm/min, corresponding to a nominal initial strain rate of 0.4 min^−1^. All the tensile tests were carried out in quintuplicate, and average values with standard deviations were reported.

#### 2.5.8. Permeability

The water vapor permeability (WVP) tests for the compression-molded films and electrospun biopapers were performed using the standardized gravimetric method ASTM E96-95. For this, Payne permeability cups from Elcometer Sprl (Hermallesous-Argenteau, Belgium) with a diameter of 3.5 cm were employed, and 5 mL of distilled water was added to each cup. The samples were secured with silicon rings, ensuring not in direct contact with water and exposed to only 100% RH on one side. The cups were then placed in a desiccator, conditioned at 0% RH and 25 °C. Then, they were weighed periodically using an analytical balance (±0.0001 g). WVP was determined by analyzing the data of weight loss over time, and the weight loss was obtained by subtracting the loss through sealing from the total loss. The permeability values were obtained by multiplying the permeance by the sample thickness. All WVP measurements were performed in triplicate.

The oxygen permeability coefficient was determined by measuring the oxygen transmission rate (OTR) using an Oxygen Permeation Analyzer M8001 from Systech Illinois (Thame, UK) at 60% RH and 25 °C in duplicate. The humidity-equilibrate samples were purged with nitrogen and then exposed to an oxygen flow of 10 mL/min. The exposure area during the test was 5 cm^2^ for each sample. The oxygen permeability (OP) was calculated taking into account the sample thickness and gas partial pressure. All OP measurements were performed in triplicate.

#### 2.5.9. Statistical Analysis

One-way analysis of variance (ANOVA) followed by a multiple comparison test (Tukey) was performed to determine the statistical differences with a significance level of 95%. The data were processed using the software OriginPro8.5 (OriginLab Corporation, Northampton, MA, USA). For all cases, *p* ≤ 0.05 was considered statistically significant.

## 3. Results

### 3.1. Solution Properties and Morphology

The solution properties and initial fiber morphology were first examined to determine whether both PHBH grades could be electrospun under the same processing conditions and to identify the annealing temperatures required for interfiber coalescence into continuous biopapers. The initial physical characteristics of the electrospinning solutions were characterized in terms of viscosity, surface tension, and electrical conductivity, as these factors have a crucial effect on the final morphology of the materials and on the stability of the electrospinning process [33]. Table 1 summarizes the properties of the two different grades of PHBH solutions, namely 151C and X131A. Both polymeric solutions exhibited similar surface tension values, below 40 mN/m, which are suitable for a stable electrospinning process [34,35,36]. However, one can observe that the X131A-grade PHBH solution had a viscosity of 1583 cP, while the 151C-grade PHBH solution had a viscosity of 2030 cP. This difference can be attributed to the varying 3HH content and potentially different molecular weights of the two grades [14]. Regarding conductivity, the solutions of 151C and X131A PHBH grades exhibited values of 1.36 ± 0.02 μS/cm and 0.54 ± 0.01 μS/cm, respectively. This could potentially be attributed to the presence of additives in the optimized grade (151C) during manufacturing [14]. Nevertheless, conductivity is low in PHAs, which is needed for better processability [27,28]. Although the two solutions showed different viscosity and conductivity values, both were electrospinable and stable under the same selected processing conditions used in this study, allowing direct comparison between the two grades.

**Figure 1:**
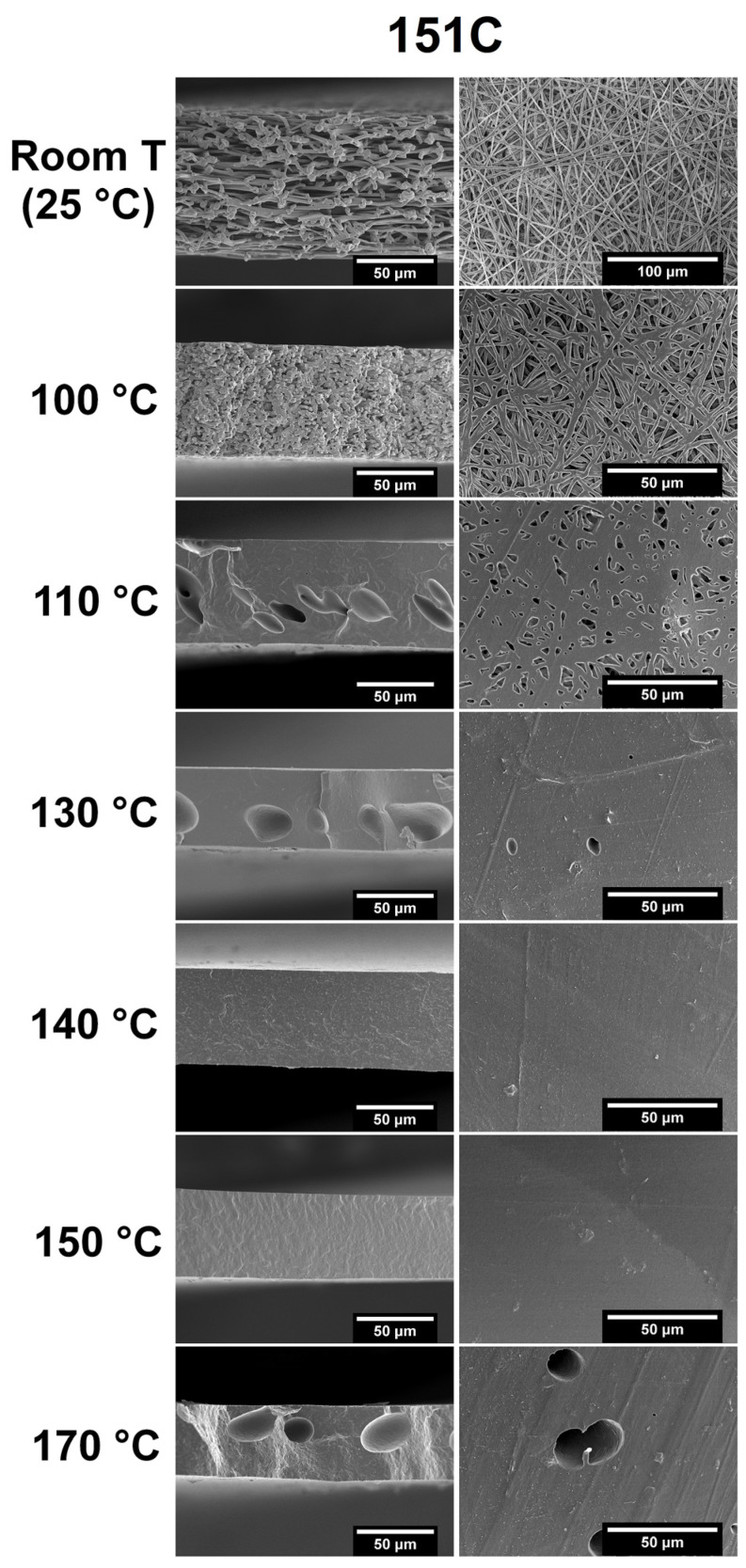
SEM images of the cross-section (**left column**), and top view (**right column**) of the electrospun 151C-grade PHBH mats without thermal post-treatment (room temperature) and annealed at 100, 110, 130, 140, 150, 170 °C for 10 s. Scale markers are 50 μm in all panels except for the room-temperature top-view image (top-right), where the scale bar corresponds to 100 μm.

**Figure 2:**
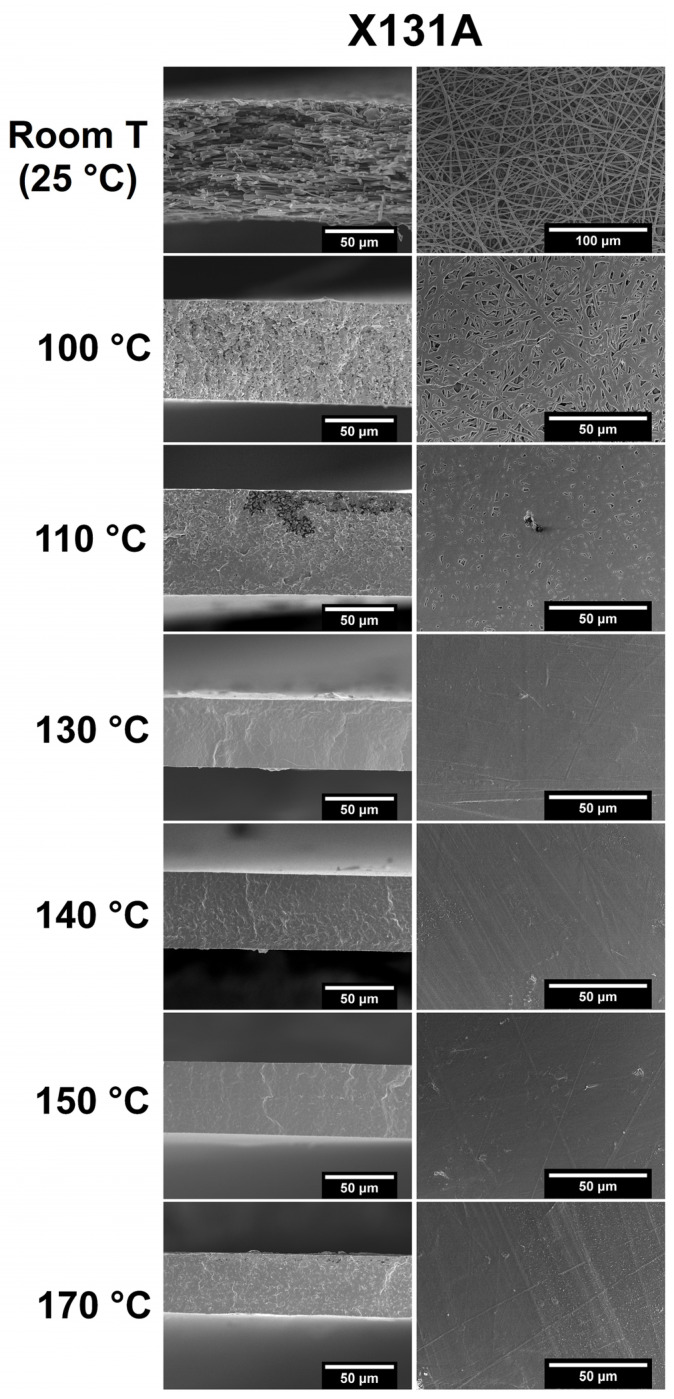
SEM images of the cross-section (**left column**), and top view (**right column**) of the electrospun X131A-grade PHBH mats without thermal post-treatment (room temperature) and annealed at 100, 110, 130, 140, 150, 170 °C for 10 s. Scale markers are 50 μm in all panels except for the room-temperature top-view image (top-right), where the scale bar corresponds to 100 μm.

Figure 1 and Figure 2 at room-temperature conditions display the morphology of the fiber mats resulting from the electrospinning process of the two distinct PHBH grades of 151C and X131A solutions, respectively. Both figures at different temperatures show the cryo-fractured cross-section surfaces (left column) and top views (right column), both before (room temperature) and after annealing. For each grade, electrospun mats without thermal post-treatment, corresponding to the figures at room temperature, produced smooth fibers without the presence of beads, with a mean fiber diameter of 2.12 ± 0.37 μm and 1.70 ± 0.38 μm, for 151C and X131A, respectively. Although the difference in mean fiber diameter was mild, this can be attributed to increased solution viscosity of 151C solution (2030 cP), which generally leads to a larger mean fiber diameter [37]. Regarding the annealing process at relatively low temperatures of 100 °C and 110 °C, the 151C and X131A PHBH fibers began to “fuse” with each other, while maintaining a high level of porosity. Importantly, the use of temperatures at 130 °C for X131A grade mats resulted in the formation of a continuous film (so-called biopapers) with extremely low porosity. As seen in the figure, this fiber coalescence occurred for 151C-grade PHBH mats at 140 °C annealing temperature. This was expected since different grades of PHBH show different melting profiles due to the variations in their 3HH content, resulting in different temperatures for fiber coalescence [27]. The aforementioned phenomenon is attributed to the compact packing/rearrangement of the electrospun fibers to minimize their surface energy upon annealing [28].

Figure 1 and Figure 2 also display the morphology of the biopapers annealed at higher temperatures (170 °C). At higher annealing temperatures, some large holes or voids were formed, which could be due to thermal degradation. Void formation was more prominent in the fiber mats of 151C, while X131A biopapers exhibited smaller holes. This phenomenon was also previously reported by Melendez-Rodriguez et al. when studying the annealing of electrospun PHBV fibers with varying 3HV content, suggesting that at temperatures higher than the selected annealing temperatures, the annealing process resulted in the formation of large voided areas [27]. Hence, in this study, the selected annealing temperatures for further characterization were 140 °C for 151C and 130 °C for X131A, respectively, defined as the lowest temperatures that yielded continuous biopapers with very low residual porosity under the fixed annealing conditions used here. The suitability of this selection was later supported by the final barrier performance of the resulting films, particularly in relation to oxygen transport.

### 3.2. Thermal Properties

**Figure 3:**
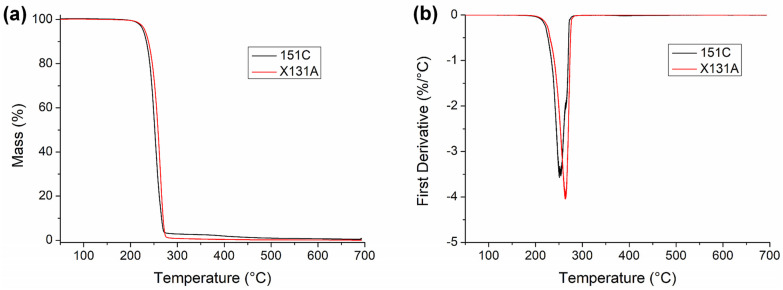
(**a**) Thermogravimetric analysis (TGA) and (**b**) first derivative (DTG) curves of the electrospun PHBH-based fibers produced from 151C and X131A grades.

Thermal analyses were carried out to verify that the selected annealing temperatures remained below the degradation regime and to position the annealing window relative to the melting behavior of each PHBH grade. In this context, the thermal stability of electrospun PHBH-based fibers produced from two different grades of 151C and X131A was first characterized by thermogravimetric analysis (TGA). The thermogravimetric curves and derivatives are presented in Figure 3, while the corresponding values of the onset degradation temperature (T_5%_), degradation temperature (T_deg_), mass loss at T_deg_, and residual mass at 700 °C are summarized in Table 2. It can be observed that both grades exhibited similar thermal stability. However, electrospun 151C fibers exhibited a slightly lower thermal stability with slightly higher residual mass at 700 °C compared to electrospun X131A fibers. Importantly, the thermal post-processing (annealing) step was performed at temperatures far below the onset degradation temperatures of both grades. The annealing temperature for 151C and X131A was 140 °C and 130 °C, respectively. Additionally, the annealing duration was also very short, lasting only 10 s. Therefore, thermal degradation during the post-processing step was avoided for both grades.

**Figure 4:**
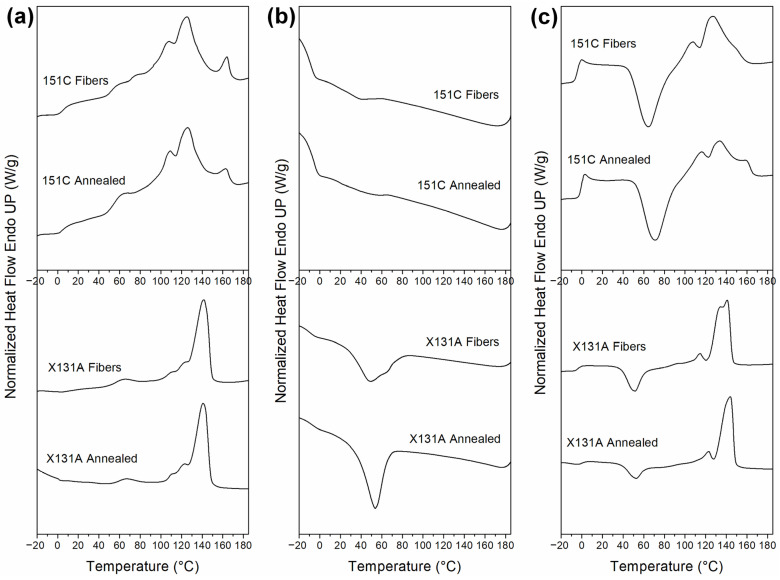
Differential scanning calorimetry (DSC) curves, (**a**) first heating, (**b**) cooling, and (**c**) second heating of the PHBH-based samples of 151C (top row), and X131A (bottom row) produced by electrospinning and annealing processes.

Next, the thermal transitions of PHBH-based samples from two different grades (151C and X131A) were analyzed by DSC. The samples from these two different grades were also compared regarding the processing methods, namely electrospinning (fibers) and annealing (biopapers). The thermal parameters of the samples during the first and second heating and cooling runs are presented in Table 3, and the corresponding thermograms are visualized in Figure 4. In the first heating run, which is more relevant for the comparison of sample form given the presence of thermal history, the thermal parameters were found to be similar in fiber and biopaper form for each grade. However, one can observe that there are distinct differences between the PHBH grades. In this regard, 151C samples displayed a complex melting behavior with multiple melting points, where the main melting peak was located at approximately 125 °C. On the other hand, X131A samples exhibited a more intense and sharper main peak at around 140 °C. These results align with the information provided by the manufacturer [38]. Additionally, both grades showed multiple melting peaks, as observed from Figure 4, which are generally attributed to the melting, recrystallization, and remelting of crystals during heating [39,40,41] or a melting process involving crystals with different lamella thicknesses and/or different crystal morphologies [42]. Notably, the 151C samples exhibited a thermogram with more distinct multiple melting peaks compared to the X131A samples, suggesting that the crystalline domains in 151C were more heterogeneous in lamella sizes and/or a lower degree of crystal perfection [43]. One reason for this can be attributed to the difference in 3HHx contents, which was higher in the 151C grade (11%) than in the X131A grade (6%), leading to the formation of more imperfect crystals due to shorter HB sequence lengths [43]. Additionally, it is worth noting that both grades exhibited a weak lowest-temperature endotherm at around 60–65 °C, just above the crystallization temperature. A comprehensive study in this regard by Hu et al. suggested that this peak corresponds to the melting of crystals formed during the primary crystallization, and that the other peaks at higher temperatures can be ascribed to the melting peak of crystals formed by recrystallization during the heating process [43].

The following cooling and second thermal runs are more closely linked to the inherent crystalline structure of the utilized materials. In the cooling run (Figure 4b), it can be seen that, regardless of the form (fiber or biopaper), 151C-based materials showed very weak and broad crystallization events. In each case, the crystallization temperature (Tc) and enthalpy (ΔHc) were not measured due to the weak corresponding signals. On the other hand, X131A-based samples presented a more intense and narrower crystallization peak, indicating easier and faster crystallization. Here, it can be said that the crystallization of PHBH is influenced by the 3HHx comonomer, requiring higher undercooling to peak as the 3HHx content increases [43].

Finally, the second thermal run was performed to gain insight into the inherent crystallinity of PHBH-based samples, and the results are presented in Figure 4c and Table 3. From Figure 4c, it is evident that both the 151C and X131A grades exhibited complex endothermic curves with multiple peaks, similar to those observed during the first heating scans; however, uniquely, the second scan revealed the presence of distinct cold crystallization events. For 151C samples, a very evident cold crystallization peak appeared at 65.1 °C and 71.4 °C, for electrospun fibers and annealed biopapers, respectively, whereas the cold crystallization enthalpies (ΔHcc) for these were similar (approximately 35 J/g). In the case of X131A samples, a cold crystallization event was milder at around 52 °C, with lower ΔHcc values of 20 J/g and 10 J/g for electrospun fibers and annealed biopapers, respectively. Cold crystallization is a well-known phenomenon commonly observed in PHAs [44]. PHBH demonstrates cold crystallization because it is unable to fully crystallize during the cooling process until reaching saturation, thus exhibiting cold crystallization in the subsequent heating scan [40]. This indicates that the samples showing cold crystallization events remain largely amorphous after the non-isothermal crystallization occurring in the cooling run [45]. Regarding this, first Di Lorenzo et al. [46], when analyzing the crystallization process of PHB, and later Barbosa et al. [47], when studying the plasticization and its influence on the thermal properties of PHBV, attributed this cold crystallization event to the rigidity of the amorphous phase, which hinders crystallization during cooling. Instead, during the subsequent heating (second heating run), the rigid amorphous phase gradually becomes mobile, leading to the occurrence of a cold crystallization event. Moreover, upon subtracting the enthalpy of cold crystallization, it becomes evident that 151C-based samples exhibit significantly lower values for enthalpy of melting (indicating lower crystallinity) when compared to X131A-based samples.

Nevertheless, the selected annealing temperatures, 130 °C for X131A and 140 °C for 151C, were within the range where endothermal events occur (see Figure 4a), indicating sufficient molecular mobility for the interfiber coalescence process to take place [27]. However, due to the complex thermal kinetics of PHAs, providing an in-depth understanding of the crystalline structure of the here-studied polymers becomes challenging [27,28]. Therefore, it is necessary to conduct further investigations employing more detailed characterization methods (see FTIR and WAXS Sections).

### 3.3. Crystalline Morphology Evaluation with Temperature—Fibers to Biopaper

#### 3.3.1. ATR-FTIR

**Figure 5:**
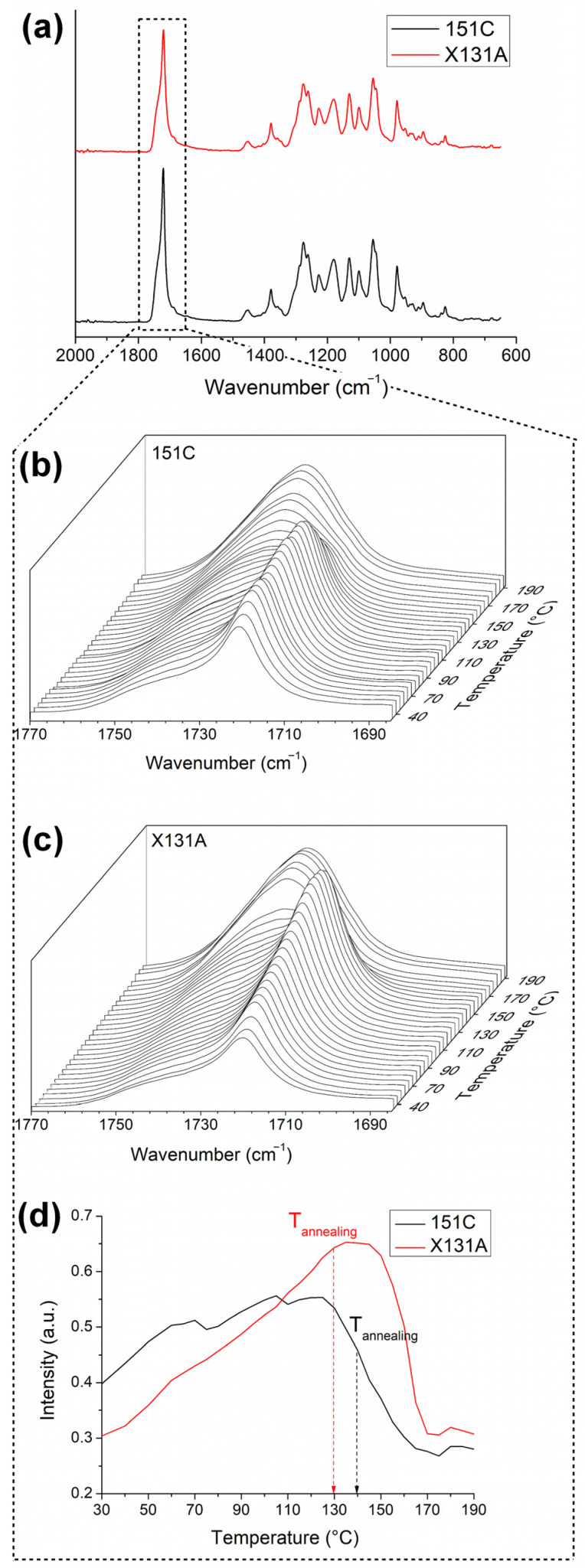
(**a**) ATR-FTIR spectra of the electrospun PHBH-based fibers taken at room temperature; evolution of the carbonyl peak at 1720 cm^−1^ with temperature for the electrospun (**b**) 151C-grade PHBH, (**c**) X131A-grade PHBH fibers, and (**d**) comparison of the evolution of the intensity of the peak at 1720 cm^−1^ with temperature for both grades.

ATR-FTIR analysis was performed to follow temperature-induced changes in local molecular order during heating and to clarify how these changes are related to interfiber coalescence during annealing. For this purpose, ATR-FTIR spectra were first collected at room temperature for the electrospun fibers produced from two different PHBH grades, 151C and X131A, and the resulting spectra are presented in Figure 5a. As expected, electrospun fibers from each grade exhibited similar characteristic peaks. The characteristic peaks of PHBH appeared at 2970, 2937, 2870, and 2850 cm^−1^, corresponding to the asymmetric stretching of C-CH_3_, and CH_2_, CH stretching, and CH stretching, respectively [48,49]. Importantly, both grades exhibited a prominent peak at 1720 cm^−1^, representing the characteristic stretching vibration of the carbonyl group (C=O). This band is sensitive to the local environment of carbonyls in ordered regions—crystalline lamellae and interfacial/mesomorphic zones—and can be used as a proxy for molecular order of a material [26,27]. Additionally, the bands at 1278, 1180, and 1049 cm^−1^ are attributed to ester backbone vibrations, and features at 1260, 1228, and 980 cm^−1^ are commonly associated with the crystalline phase of PHBH [48]. Here, vibrational spectroscopy can offer information about the molecular conformation within the polymer backbones, particularly in ordered/crystalline regions. However, it is important to note that this technique is not exclusively limited to crystals, as it lacks sensitivity to the lateral arrangement necessary for crystallinity [27]. Therefore, further investigation was conducted to evaluate the changes in the molecular order of PHBH-based fibers by examining the carbonyl band at 1720 cm^−1^ as a function of temperature.

Figure 5b,c present the evolution of the aforementioned carbonyl peak at 1720 cm^−1^ with temperature for the electrospun fibers produced from grades 151C and X131A, while Figure 5d illustrates the intensity of this peak. Overall, both grades showed a two-stage evolution: the 1720 cm^−1^ band intensity initially increased at sub-melting temperatures and then decreased as the material entered the melting transition, while a broader band around ~1740 cm^−1^ became more pronounced. It was suggested previously by Sato et al., when studying the C=O stretching region of P(HB-co-HV) by FTIR, that these peaks are associated with the crystalline and amorphous phases of PHAs, respectively [50]. Specifically, electrospun 151C fibers exhibited an increase in intensity of the carbonyl peak at 1720 cm^−1^ until reaching about 130 °C (Figure 5b,d). It is worth noting that this increase was not continuous, as there are slight, inconsistent decreases observed at around 70 °C and 110 °C, followed by subsequent increases. However, after 130 °C, a more significant and continuous drop in intensity occurred. On the other hand, the trend in intensity alteration for X131A fibers was similar but with notable differences. In this case, the increase in intensity of the carbonyl peak (1720 cm^−1^) was more steady and steep (Figure 5c,d) until reaching a temperature of around 140 °C. After this temperature, similar to 151C fibers but more rapidly, a progressive decrease occurred until the peak disappears.

The observed intensity increase at the lower temperatures (up to 130 °C for 151C and 140 °C for X131A) suggests thermally induced molecular ordering/perfection in the carbonyl environment. This behavior matches electrospun PHBV mats, where the 1720 cm^−1^ band increases at low T and then decreases as the material enters the melting transition; the decrease is attributed to a progressive loss of molecular order as thin/defective lamellae melt first, followed by more robust crystals [27]. As shown above, the evolution of the carbonyl peak (1720 cm^−1^) for 151C was mild, while the alterations for X131A were more pronounced and steeper. From this, it is possible to hypothesize that 151C fibers may have a broader distribution of ill-defined crystal lamellae and/or less consistent local order, while X131A fibers exhibit a more monomodal and coherent ordered region. This can also be inferred from the DSC curves, with 151C exhibiting multiple peaks, in contrast to the X131A curves (Figure 4). Ultimately, the selected annealing temperature of 130 °C for X131A fibers lies close to the broad intensity maximum, beyond which the molecular order begins to progressively decrease. For the case of 151C fibers, the selected annealing temperature (140 °C) falls within the regime where the intensity was already decreasing (see Figure 5d). These results indicate that fiber coalescence was grade-dependent: in X131A samples, interfiber coalescence was more strongly associated with thermally induced molecular ordering, whereas in 151C it was predominantly linked to molecular disorder arising from partial melting of thin/ill-defined lamellae.

#### 3.3.2. Crystalline Morphology

**Figure 6:**
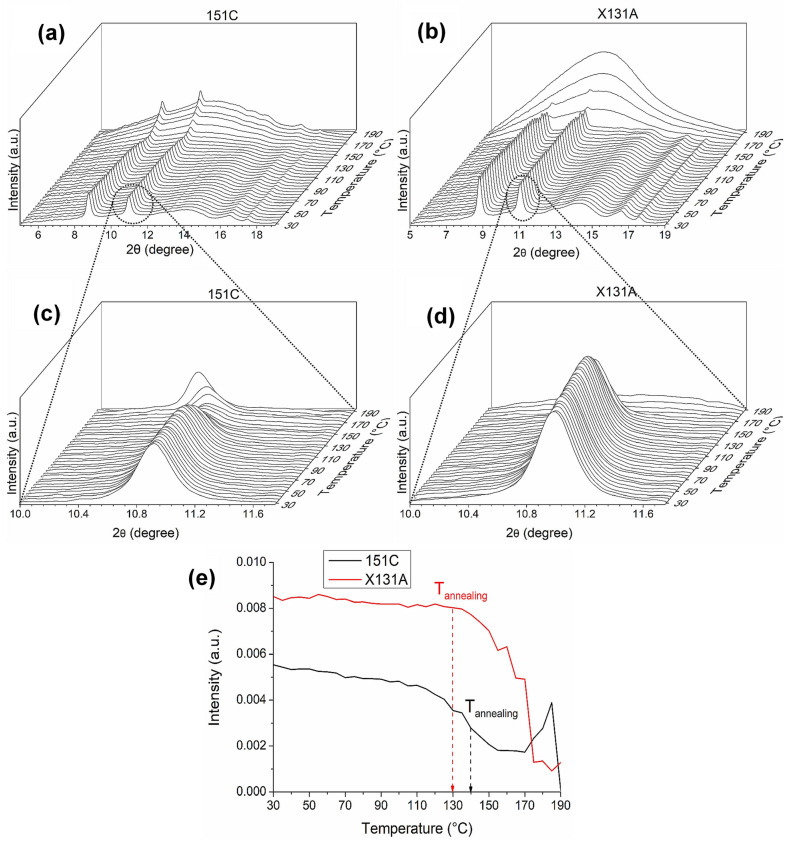
Wide-angle X-ray scattering (WAXS) patterns of electrospun (**a**) 151C and (**b**) X131A grades of PHBH fibers with a heating ramp from 30 °C to 190 °C. WAXS patterns zoomed around 11° peak for the electrospun (**c**) 151C, and (**d**) X131A grades of PHBH fibers, and (**e**) evaluation in relative intensity of the 11° peak of these diffractograms.

As complementary characterizations to ATR-FTIR analysis as a function of temperature, simultaneous time-resolved WAXS and SAXS experiments were performed using synchrotron radiation at ALBA synchrotron facilities (Barcelona, Spain) to investigate the crystallinity and phase morphology of electrospun PHBH-based fibers in function of temperature. Figure 6a,b depict the WAXS diffractograms of the electrospun fibers for the 151C and X131A grades, respectively, during the heating ramp from 30 °C to 190 °C. Both grades of PHBH exhibited the typical crystalline structure of pure PHB homopolymer. For each grade, the characteristic peaks corresponding to the (020) and (110) diffractions were revealed at 8.7° and 10.9°, respectively [51]. Additional diffraction peaks with lower intensities were also observed at higher 2θ values. For instance, the peak located in the range of 12° to 16° corresponds to deconvoluted peaks of (021), (101), and (111) diffractions, while the peaks at 16.5° and 17.5° are attributed to the (121) and (040) diffractions, respectively [27,52]. It is worth noting that 3-hydroxyhexanoate (3HHx) comonomers are non-crystallizable; thus, regardless of the 3HH content present in 151C and X131A grades, the WAXS diffractograms of these biopolymers follow the ones of pure PHB homopolymer without the presence of 3HH lattice crystals [53,54]. From Figure 6a,b, one can observe that 151C fibers exhibited an increase in amorphous halo after reaching a temperature of approximately 155 °C, while for X131A fibers, this occurred around 170 °C. During this event, the intensity of the crystalline peaks progressively diminished for both cases. However, unlike the X131A diffractogram, the crystalline peaks of 151C fibers were somewhat more persistent, continuing to appear at higher temperatures despite the increasing amorphous halo. In this regard, Figure 6c,d provide a close-up view of the characteristic crystalline peak corresponding to the (110) plane during the heating ramp, while Figure 6e plots the relative intensity of this peak for both biopolymer grades as temperature increases. From Figure 6e, it can be seen that the intensity of the crystalline peak remained constant until 140 °C for X131A fibers, beyond which a sharp intensity drop occurred, consistent with the major melting event. However, this trend was different for 151C fibers, which showed a slight decrease until 120 °C, after which the downward slope steepened until 170 °C. Subsequently, a notable intensity increase occurred until 185 °C, and ultimately resulted in complete melting. It is worth noting here that during the intensity increase until 185 °C, the crystalline peak shifted to lower 2θ values, indicating an alteration in the crystalline structure. Overall, no classical premelting intensity build-up in the crystalline peak was observed for both 151C and X131A fibers during the thermal ramp. Such an intensity increase upon heating towards the melting point is commonly reported for many PHAs (e.g., PHBV) [27,28]. This behavior seems to be different for PHBH fiber samples, for which the intensity of the (110) crystalline lattice peak remained constant for X131A fibers, while 151C fibers exhibited only a slight decrease, as the temperature increased. In this regard, Nguyen et al. [55] reported that higher 3HH content slows nucleation and crystallization and identified metastable “intermediate structures” whose persistence increases with 3HH, delaying their conversion to lamellae and thereby retarding crystallization kinetics and crystalline perfection. This mechanistic picture may explain the absence of a premelting WAXS-intensity build-up observed here. Additionally, a similar behavior was also observed by Ye et al. in their in situ temperature-dependent WAXS experiments on microbially produced PHBH polymer with 12% 3HH content [53]. In their case, the intensity of (020) and (110) diffraction planes slightly decreased as the temperature approached the melting point of the polymer, and within a small temperature range (118 °C to 126 °C), immediately before the melting point, the intensities of the planes remained constant. After that, a further increase in temperature resulted in a sudden drop in the diffraction peaks due to the melting.

Synchrotron WAXS observations exhibit some correlation with the results obtained from DSC. For instance, X131A fibers demonstrated a main melting event at approximately 142 °C (see Figure 4a). However, 151C fibers presented a more complex melting behavior with multiple melting peaks, where the main melting point was at approximately 125 °C (Figure 4a). As can be seen from Figure 6e, these main melting events correspond to the temperature at which the intensity of the crystalline peak begins to drop. Beyond this broad WAXS-DSC agreement, WAXS and temperature-evolving ATR-FTIR highlight different aspects of the structural evolution with temperature. In ATR-FTIR, as the temperature increased, the intensity of the carbonyl peak initially increased at sub-melting temperatures, indicating a gradual enhancement in molecular order. This increase was subsequently followed by a more pronounced drop in intensity as the material entered the melting transition. Conversely, in the synchrotron WAXS experiments, the intensity of the (110) diffraction plane for X131A fibers remained essentially constant until the final drop, whereas for 151C it exhibited a mild, progressive decrease already at lower temperatures. The main difference between the two techniques, temperature-dependent FTIR and synchrotron WAXS, is that the FTIR data can detect ordered chain segments along the polymer backbone, regardless of their presence within crystals or lateral arrangement. In contrast, WAXS demonstrates sensitivity towards lateral arrangement, namely crystallinity [27]. Consequently, interfiber coalescence in PHBH appears to be controlled by the interplay of thermally induced local ordering at sub-melting temperatures and the earlier onset of molecular disorder/premelting of thinner, less-perfect lamellae, with the relative contribution being grade-dependent. Importantly, coalescence occurred prior to complete melting (loss of WAXS crystallinity), where sub-melting ordering and incipient melting of less-perfect lamellae jointly facilitate coalescence.

**Figure 7:**
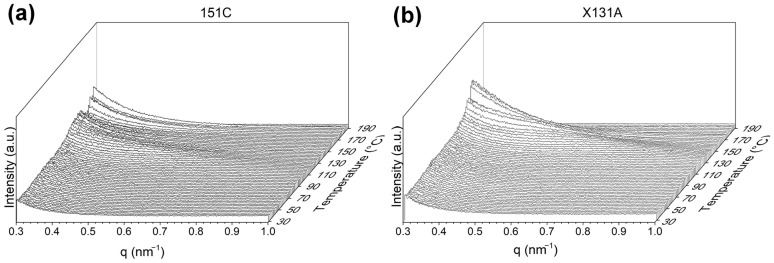
Small-angle X-ray scattering (SAXS) patterns of (**a**) 151C and (**b**) X131A grades of PHBH fibers with a heating ramp from 30 °C to 190 °C.

Figure 7 shows the temperature evolution of SAXS patterns for 151C and X131A PHBH fibers throughout a thermal ramp ranging from 30 °C to 190 °C, which are sensitive to the PHBH long period [51]. As observed in Figure 7, the intensity of SAXS peaks increased with rising temperature for both cases. Furthermore, beyond 130 °C, the peaks tended to shift towards lower q values, indicating an expansion in the long period. This behavior is commonly observed in semicrystalline polymers, where the repeat unit tends to expand before the melting process [27]. It is also worth noting that these results correlate with the changes observed in temperature-dependent FTIR results (refer to Figure 5).

### 3.4. Evaluation of PHBH-Based Samples Produced by Different Methods

In this section, the films and biopapers obtained via compression molding (as a conventional technique) and annealing, respectively, are compared in terms of optical, crystalline, mechanical, and barrier properties of importance in food packaging applications. Additional information regarding basic characterizations, including SEM and DSC, of the compression-molded films can be found in Supplementary Materials.

#### 3.4.1. Optical Properties

**Figure 8:**
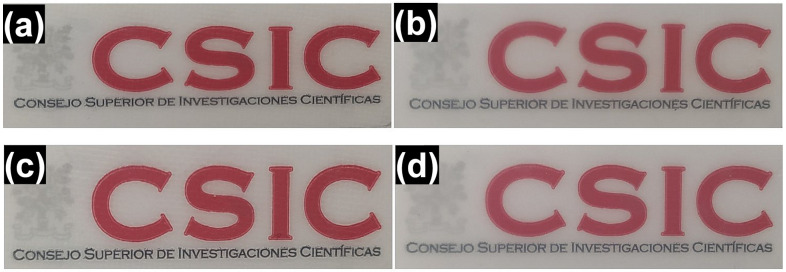
Background transparency pictures of PHBH-based samples: (**a**) annealed 151C, (**b**) compression-molded 151C, (**c**) annealed X131A, (**d**) compression-molded X131A.

Optical measurements were carried out to evaluate the visual appearance and transparency of the annealed biopapers in comparison with compression-molded films for packaging applications. Figure 8 shows the visual appearance of PHBH biopapers and films from two different grades (151C and X131A) prepared by annealing and compression molding methods. One can observe that the samples produced by the annealing method (Figure 8a,c) have a clearer visual appearance compared to compression-molded samples (Figure 8b,d). This can be indirectly attributed to the lower crystallinity observed in annealed samples. Additionally, their lower thicknesses (80 μm) compared to compression-molded samples (120 μm) may contribute to enhanced visual clarity. In any case, no color was detected for all samples, indicating that they were not thermally abused [28].

To quantify these visual differences, contact transparency and opacity parameters were measured and recorded in Table 4. All samples showed similar contact transparency, while compression-molded X131A PHBH exhibited a slightly higher value. On the other hand, both annealed 151C and X131A biopapers demonstrated similar opacity values of 0.01 ± 0.00 mm. However, the opacity of the compression-molded samples was found to be higher than that of the annealed ones. The opacity of compression-molded 151C and X131A films increased to 0.03 ± 0.01 mm and 0.07 ± 0.01 mm, respectively. Overall, the annealed PHBH samples exhibited lower opacity than compression-molded films, indicating higher optical clarity. Contact transparency was comparable across samples, which can be advantageous for food packaging applications.

#### 3.4.2. Crystallinity

**Figure 9:**
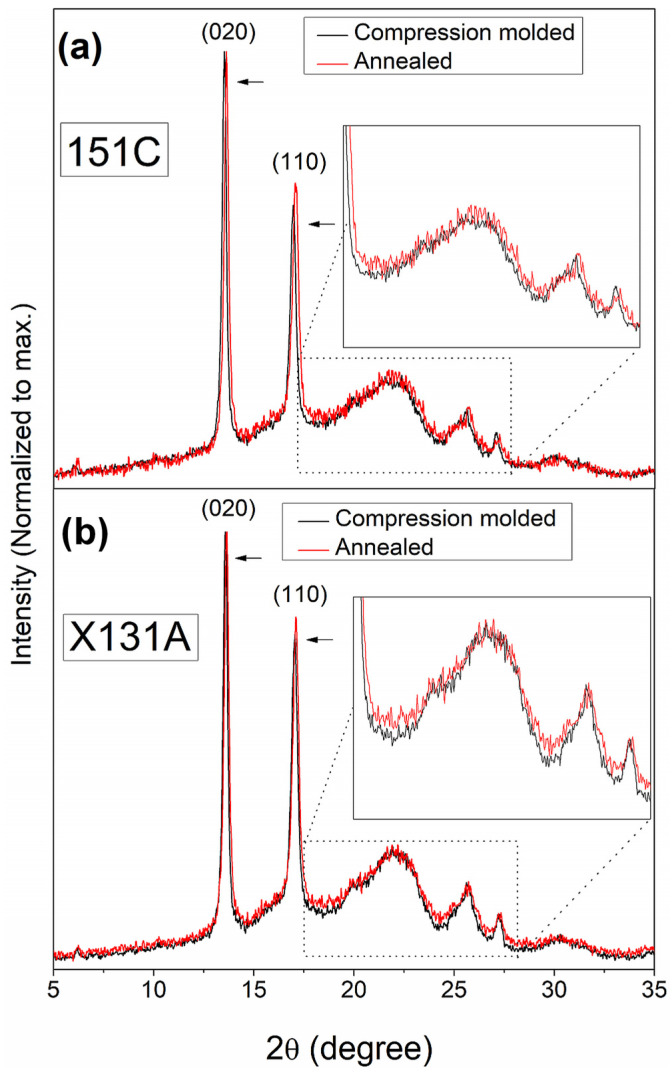
Wide-angle X-ray scattering (WAXS) patterns of (**a**) 151C, and (**b**) X131A grades of PHBH samples produced by compression molding and annealing methods. Black lines correspond to compression-molded films, and red lines correspond to annealed biopapers. The insets show enlarged views of the diffraction patterns in the 17–28° range. The arrows indicate the slight shift of the crystalline peaks toward lower angles for the compression-molded samples.

Conventional WAXS analysis was used to compare the crystalline structure and estimated crystallinity of the final biopapers and compression-molded films after processing. Accordingly, room-temperature WAXS experiments were conducted on PHBH-based samples (151C and X131A grades), comparing films obtained via compression molding and biopapers obtained via annealing. The corresponding WAXS diffraction patterns are presented in Figure 9 within the 2θ range of 5° to 35°, with inset figures zooming to the range of 17° to 28°. As discussed in the previous section (see Section 3.3.2.), PHBH copolymers, which contain non-crystallizable 3-hydroxyhexanoate (3HH) comonomers, exhibit a PHB-like crystalline structure [39,53,54], as shown in Figure 9. All samples displayed an orthorhombic crystalline lattice with a space group of P212121 (D24) [21,39]. Additionally, the most characteristic peaks of pure PHB homopolymer were observed at around 13.7° and 17.1°, corresponding to the (020) and (110) diffractions, respectively. Comparing the PHBH grades, X131A-based samples exhibited higher intensities in crystalline peaks (Figure 9b) compared to the crystalline peak intensities of 151C-based samples (Figure 9a), whose difference is more evident when comparing the inset figures. Therefore, it can be expected that the crystallinity for 151C samples may be lower than that of X131A samples. Furthermore, when comparing the film preparation methods, biopapers produced via the annealing method (Figure 9, red lines) exhibited a slightly more pronounced amorphous halo than films produced via compression molding (Figure 9, black lines), as more clearly seen in the inset, this difference was more evident for X131A samples. These variations have been previously attributed to the inherently reduced crystallinity resulting from the electrospinning process [26,28]. Another interesting observation is that PHBH films produced via compression molding displayed a slight shift in the crystalline peaks towards lower angles (Figure 9, arrows), suggesting an alteration in the crystal structure of PHBH-based films caused by the production methods.

Next, the unit-cell parameters (a, b, and c) of the PHB-like lattice, crystallinity, and interplanar distance of the PHBH-based films and biopapers were estimated, and the results are presented in Table 5. The table shows that both grades of PHBH samples, 151C and X131A, showed slightly higher estimated PHB-like lattice parameters for compression-molded films. Additionally, Table 5 displays the estimated degree of crystallinity obtained from the diffractograms in Figure 9. To the best of the knowledge available in the literature, PHBHs generally exhibit lower crystallinity compared to the homopolymer PHB, which typically has a degree of crystallinity higher than 60–65% [21,56,57]. Furthermore, according to the manufacturer, X131A-grade PHBH is a rigid type, while 151C is more flexible [38]. Hence, it is anticipated that X131A-grade PHBH samples exhibit higher crystallinity, whereas 151C-grade samples are expected to display lower crystallinity. In line with this expectation, the annealed X131A-grade PHBH biopapers exhibited an estimated crystallinity of 41%, which was lower for the 151C biopapers with 35% crystallinity (Table 5). Comparing the film production methods of compression molding and annealing, it can be observed from the table that both grades showed differences in estimated crystallinity. Compared to the biopapers obtained through annealing, the compression-molded 151C films exhibited a 3% increase, whereas the compression-molded X131A films showed a 5% increase. This can be attributed to the higher thermal stress and longer duration applied to the compression-molded samples compared to the annealed ones. It is known that PHAs can develop higher crystallinity at higher post-processing temperatures and longer durations [58,59]. Furthermore, both annealed grades showed similar interplanar distances, with slightly higher estimated values observed for the samples produced by the compression molding method. Therefore, these results suggest that the crystalline morphology of PHBH-based materials, as reflected by unit-cell parameters, crystallinity, and interplanar distance, may exhibit a response to external post-processing factors such as temperature and time.

#### 3.4.3. Mechanical Properties

Mechanical testing was performed to determine how the electrospinning-plus-annealing route influences stiffness, strength, and ductility relative to conventional compression molding. The main mechanical properties of PHBH-based samples are summarized in Table 6, including the elastic modulus (E), tensile strength at break (σ_b_), elongation at break (ɛ_b_), and toughness. As an overall interpretation of the table, one can notice that X131A grade samples exhibited more rigid behavior than 151C grade samples in all forms, as suggested by the manufacturer [38]. As a comparison of the grades in fiber form, electrospun 151C fibers exhibited an E, σ_b_, and ɛ_b_ values of 512 MPa, 7.8 MPa, and 70.4%, whereas these were 528 MPa, 6.3 MPa, and 43.2% for X131A fibers, respectively. From this, it can be seen that the main mechanical difference occurred in elongation at break, while E and σ_b_ values were approximately the same. The mechanical performance of PHBH fibers was reported previously by Tanaka et al. [60]. In their study, it was reported that PHBH utilized had a 3HH content of 5.5 mol%, equivalent to the 3HH content found in the X131A grade, and PHBH fibers with an average diameter of 543 nm were generated through the electrospinning technique using a solution containing 2 wt.% PHBH dissolved in hexafluoroisopropanol (HFIP). The Young’s modulus, tensile strength, and elongation at break values were reported as 291.5 MPa, 8 MPa, and 61.5%, respectively, suggesting more flexible fibers compared to X131A fibers reported in our study. However, it is worth noting that the mechanical performance of electrospun fibers is well-known to be dependent on electrospinning solution and process parameters, as well as the morphology [61,62,63]. Hence, a more accurate comparison could be made in the continuous film form.

For the purposes of comparison, the two film production techniques were analyzed. According to Table 6, annealed 151C biopapers showed E, σ_b_, and ɛ_b_ values of 1353 MPa, 25.1 MPa, and 14.1%, respectively, whereas these values were 1890 MPa, 26.8 MPa, and 2.5% for the annealed X131A biopapers, respectively. As observed in both the fibrous forms of the two grades and as expected, the 151C grade exhibited more flexible behavior compared to X131A, even in the film form. However, when both grades are produced via compression molding, the mechanical parameters were found to be slightly different compared to those produced via annealing, with no clear trend. When 151C film was produced via compression molding, the films experienced a slight decrease in elastic modulus of 1122 MPa, whereas no significant differences were observed in σ_b_, and ɛ_b_ parameters. Similar behavior was also observed for the compression-molded X131A films, which exhibited a decrease in modulus (1692 MPa) and a slight, though not significant, increase in tensile strength and elongation at break values compared to the annealed counterparts (Table 6). Although the mechanical properties did not vary significantly between the films produced by different methods, the observed slight differences contrast with some extent of the previous reports. For instance, Cherpinski et al., in their study on optimizing the annealing process to obtain continuous electrospun PHB biopapers, suggested that annealed electrospun biopapers demonstrate higher elongation at break, while mechanical strength remains similar compared to compression-molded films [26]. Furthermore, as reported by Alp-Erbay et al. [64] and demonstrated by Melendez-Rodriguez et al. [28], higher mechanical flexibility with slightly lower mechanical strength can be obtained in the annealed electrospun biopapers due to the unique structure of coalesced and rearranged electrospun fibers, which have interactions in between, such as point bonding, fiber slipping over one another, and alignment. However, it is important to note that the mechanical properties are highly dependent on the possible surface and/or internal defects occurring during the process. These defects may play a role in causing decreased mechanical strength [65,66]. In any case, all developed PHBH-based films and biopapers showed sufficient mechanical characteristics to be used in packaging applications [14].

As a sustainable packaging material, PHBH is a strong candidate with enhanced mechanical properties and processability compared to PHB homopolymer or even PHBV copolymer [9]. In this regard, researchers, as we do in this study, are still endeavoring to unveil the possible advantages of PHBH to be used in sustainable packaging applications. For instance, Schmid et al. produced X131A films by injection molding [67]. According to their reports, X131A films exhibited a mechanical performance similar to the results presented here, with E, σy, and ɛb values of 1550 MPa, 29.9 MPa, and 5.03%, respectively. However, it is worth noting that they did not specify when the mechanical tests were performed. Indicating the time scale is of great importance since PHAs undergo progressive embrittlement over time due to well-known phenomena such as secondary crystallization and physical aging of the amorphous phase [39]. In this regard, Feijoo et al. developed cast extruded films, and the mechanical tests were performed 15 days after the sample preparation [39]. For this, they used PHBH with 11-% mol 3HH content (X151A-grade, Kaneka), which has a similar 3HH content to the 151C-grade PHBH used in this study. After 15 days, their PHBH films showed a mechanical behavior of 1326 MPa in elastic modulus, 24.3 MPa in yield strength, and 12.7% in elongation at break. Interestingly, it can be observed that 151C films with elongation at break values of approximately 12–14% (Table 6) exhibited somewhat higher elastic modulus and tensile strength than other PHA counterparts with similar elongation at break values [14,27,68].

Finally, differences in the mechanical properties between PHBH and its homopolymer, PHB, should also be mentioned. For instance, Cherpinski et al. developed electrospun PHB biopapers through an annealing process, and E, σ_y_, and ɛ_b_ values were reported as 1104 MPa, 17.8 MPa, 2.9%, respectively [26]. Furthermore, another study reported the mechanical performance of electrospun biopapers produced from a commercial copolyester, PHBV (2 mol% of 3HV content, ENMAT Y1000P), with E, σ_y_, and ɛ_b_ values of 1252 MPa, 18.1 MPa, and 2.4%, respectively [69]. These comparative mechanical assessments reveal that the mechanical performance of the X131A PHBH grade closely resembles that of PHB and PHBV. Conversely, the 151C PHBH grade exhibits notably enhanced ductility, setting it apart from its counterparts.

#### 3.4.4. Barrier Properties

Barrier measurements were carried out to assess whether the electrospun biopapers provide water vapor and oxygen barrier performance relevant to packaging, and to compare these properties with those of conventionally processed PHBH films. The water vapor (WVP) and oxygen (OP) permeability values for the 151C and X131A grades of PHBH films and biopapers are presented in Table 7. It can be observed that the electrospun biopapers obtained through the annealing method exhibited WVP values of 0.77 × 10^−14^ kg·m·m^−2^·Pa^−1^·s^−1^ and 0.58 × 10^−14^ kg·m·m^−2^·Pa^−1^·s^−1^ for 151C and X131A, respectively. In the case of the compression-molded films, these were slightly lower, showing WVP values of 0.51 × 10^−14^ kg·m·m^−2^·Pa^−1^·s^−1^ and 0.33 × 10^−14^ kg·m·m^−2^·Pa^−1^·s^−1^ for 151C and X131A, respectively. A similar trend can also be observed in oxygen permeability values. The OP values were found to be 14.26 × 10^−19^ m^3^·m·m^−2^·Pa^−1^·s^−1^ and 8.77 × 10^−19^ m^3^·m·m^−2^·Pa^−1^·s^−1^ for electrospun 151C and X131A biopapers, respectively. In the case of the compression-molded films, the OP values were 11.52 × 10^−19^ m^3^·m·m^−2^·Pa^−1^·s^−1^ and 6.35 × 10^−19^ m^3^·m·m^−2^·Pa^−1^·s^−1^, following the same respective order. These differences in barrier properties between annealed biopapers and compression-molded films were expected, as the barrier performance is directly related to the porosity within the films, the molecular order, and the crystallinity content matured in the copolyester [26,27,28]. In general, a high barrier property can be obtained when the barrier material has lower porosity, higher crystallinity, and improved molecular order. As demonstrated in this study, the films produced via the compression-molded method exhibited an increased crystallinity (see Section 3.4.2., Table 5). As reported for semicrystalline polymers, crystallinity is a key factor governing permeability, since polymer crystals are essentially impermeable, increase the tortuosity of the diffusion path, and restrict the mobility of the amorphous phase [70]. Furthermore, the formation of annealed biopapers occurred by means of fiber coalescence, resulting in a specific fiber alignment. As a consequence of their fibrillar nature, biopapers may possess some level of porosity [26,28]. Hence, it was expected that compression-molded films would exhibit higher barrier performance compared to the biopapers produced through annealing. Comparing the PHBH grades, 151C samples showed more flexibility, and lower WAXS crystallinity and molecular order compared to the X131A counterpart samples. This could potentially explain why the 151C samples demonstrated the highest permeability to water vapor and oxygen across all forms.

Barrier performance serves as a critical and highly significant parameter for the practical utilization of food packaging. In this regard, PHBH has demonstrated its potential and continues to be investigated due to its superior barrier performance compared to the majority of polymers [9].

To compare the here-prepared PHBH-based samples with other PHBH counterparts, Vandewijngaarden et al. prepared PHBH (10.5 mol% of 3HH content) films via compression molding and analyzed the barrier properties two weeks after the sample preparation at 23 °C and 0% RH [71]. According to the results, the PHBH films exhibited an OP value of 9.49 × 10^−19^ m^3^·m·m^−2^·Pa^−1^·s^−1^. In another study by Zhou et al., the OP values of a solvent-cast PHBH (11 mol% of 3HH content) films were reported 9.75 × 10^−19^ m^3^·m·m^−2^·Pa^−1^·s^−1^, measured at room temperature; the RH was not specified [49]. As another solvent-cast PHBH (11 mol% of 3HH content) film, Qiu et al. reported an OP value of 14.12 × 10^−19^ m^3^·m·m^−2^·Pa^−1^·s^−1^, measured according to ASTM D1434, although the exact test conditions were not specified [72]. Finally, in a study by Beukelaer et al., two different grades of PHBH, X131A (6 mol% of 3HH content) and X151A (11 mol% of 3HH content) were produced using the injection molding method, and barrier properties of the samples were reported as water vapor transmission rate (WVTR) at 23 °C and 85% RH and oxygen transmission rate (OTR) at 25 °C and 50% RH [14]. According to their results, X131A films exhibited WVTR and OTR levels of 7.9 g·m^−2^·day^−1^, and 63 mL·m^−2^·day^−1^·bar^−1^, respectively. Furthermore, the barrier performance of a PHBH grade with a higher 3HH content was lower, showing similar percentage differences as we observed in this study. The WVTR and OTR levels of PHBH with 11 mol% 3HH were reported as 11.6 g·m^−2^·day^−1^ and 114 mL·m^−2^·day^−1^·bar^−1^, respectively. Overall, it can be observed that the barrier performance of the PHBH-based films reported here is in good agreement with the literature.

When comparing PHBH-based films with their PHA counterparts, one should first assess the barrier performance of the homopolymer, PHB. In this sense, Cherpinski et al. reported a WVP value for the electrospun PHB biopapers as 0.52 × 10^−14^ kg·m·m^−2^·Pa^−1^·s^−1^, measured at 25 °C using the ASTM gravimetric method under a 100–0% RH gradient [26]. Furthermore, in their study, it was suggested that the lowest permeability values were obtained for the continuous compression-molded PHB films, which was attributed to the sample compactness and molecular order. When comparing the PHBH with other PHA counterparts, it is interesting to observe that the commercial poly(3-hydroxybutyrate-co-3-hydroxyvalerate (PHBV) with the lowest 3-hydroxyvalerate (3HV) content (2 mol%) was found to have superior barrier properties against oxygen but inferior against water vapor. For instance, Melendez-Rodriguez et al. reported WVP and OP values for the electrospun commercial PHBV biopapers as 5.34 × 10^−14^ kg·m·m^−2^·Pa^−1^·s^−1^ and 0.37 × 10^−19^ m^3^·m·m^−2^·Pa^−1^·s^−1^, respectively, measured for WVP at 25 °C using the ASTM E96-95 gravimetric method under a 100–0% RH gradient and for OP at 25 °C and 60% RH [69]. Next, the same author also investigated the barrier performance of the biopapers obtained via annealing cheese whey-derived PHBV polyesters with different 3HV content of 20%, 40%, and 60% [27]. According to the mechanical performance, the most interesting sample was PHBV with 40 mol% 3HV, and its WVP and OP values were reported as 0.8 × 10^−14^ kg·m·m^−2^·Pa^−1^·s^−1^ and 7.4 × 10^−19^ m^3^·m·m^−2^·Pa^−1^·s^−1^, respectively, these being also measured for WVP at 25 °C under a 100–0% RH gradient and for OP at 25 °C and 60% RH, which is close to the here-in reported values.

Barrier performance serves as a critical and highly significant parameter for the practical utilization of food packaging. The current state of the art for packaging products is dominated by polyethylene (PE) and polypropylene (PP) due to their desirable barrier properties to water, while polyethylene terephthalate (PET) stands for superior barrier to oxygen [14]. When other common plastic packaging materials such as polystyrene (PS) and polyvinyl chloride (PVC) are included, they account for more than 90% of the total volume of plastics used in the industry, consisting of non-biodegradable plastics [73]. In this context, PHBH represents a promising alternative to these polymers thanks to its comparable barrier properties, tailorable mechanical properties, processing behavior similar to other polymers, and, most importantly, its biobased nature and excellent biodegradability in soil, marine, and freshwater [9]. Hence, this study highlights the potential of combining electrospinning with a mild annealing post-processing step conducted below complete melting. This synergistic approach has demonstrated the ability to create exceptional films—so-called biopapers—with well-balanced properties, compared to conventional high-temperature film production methods such as compression molding.

## 4. Conclusions

The present study developed electrospun poly(3-hydroxybutyrate-co-3-hydroxyhexanoate) (PHBH) biopapers and evaluated their potential for packaging applications. Two commercial PHBH grades (151C and X131A) were electrospun into fiber mats (average fiber diameter ~2 μm) and converted into continuous biopapers by brief annealing. Selected annealing temperatures of 140 °C (151C) and 130 °C (X131A) enabled interfiber coalescence while avoiding complete melting. Thermal analysis showed that both grades remained stable up to 227–231 °C, with maximum degradation temperatures of 256–265 °C. DSC revealed a complex melting behavior for 151C (multiple melting peaks with a maximum around 125 °C), whereas X131A showed a single melting peak with a maximum around 140 °C. The structural mechanism of interfiber coalescence was investigated using time-resolved synchrotron SAXS/WAXS together with temperature-dependent FTIR spectroscopy. The results indicated that interfiber coalescence occurs within the melting-transition (premelting) regime prior to complete melting and is governed by an interplay between thermally induced local ordering at sub-melting temperatures and incipient premelting/partial melting of thin, ill-defined lamellae, with a grade-dependent balance between these contributions.

Finally, electrospun biopapers were compared with compression-molded PHBH films. X131A samples exhibited higher crystallinity than 151C, and compression-molded films were 3–5% more crystalline than electrospun biopapers. The annealed biopapers showed higher transparency and greater ductility than compression-molded films, while 151C showed the most ductile behavior (elongation at break 14.1% vs. 2.5% for X131A). In barrier testing, X131A biopapers exhibited lower WVP and OP than 151C biopapers (0.58 × 10^−14^ vs. 0.77 × 10^−14^ kg·m·m^−2^·Pa^−1^·s^−1^; 8.77 × 10^−19^ vs. 14.26 × 10^−19^ m^3^·m·m^−2^·Pa^−1^·s^−1^), while biopapers showed slightly higher permeabilities than compression-molded films, in line with their lower crystallinity and electrospun-derived structure.

Overall, electrospinning followed by mild annealing provides PHBH biopapers with a balanced combination of optical, mechanical, and barrier performance in the same practical range as conventional melt-processing films, while enabling thinner films under substantially lower thermal load. In this sense, the route is particularly attractive for PHBH, as well as for other biopolymers with narrow processing windows, and may also be advantageous when thermolabile functional ingredients are to be incorporated. Therefore, beyond proving insight into the electrospun-to-film transformation of PHBH systems, this study supports the technological relevance of the electrospinning-plus-annealing approach as a complementary processing route for PHBH-based packaging applications.

## Figures and Tables

**Table 1:** Properties of the polymeric solutions of two different grades (151C, X131A) of PHBH.

Solution	Viscosity (cP)	Surface Tension (mN/m)	Conductivity (μS/cm)	Fiber Diameter (μm)
151C	2030 ± 6 ^a^	23.7 ± 0.2 ^a^	1.36 ± 0.02 ^a^	2.12 ± 0.37 ^a^
X131A	1583 ± 5 ^b^	23.2 ± 0.1 ^a^	0.54 ± 0.01 ^b^	1.70 ± 0.38 ^a^

^a,b^ Different letters in the same column indicate a significant difference among the samples (*p* < 0.05). Values of viscosity, surface tension, and conductivity are given as mean ± standard deviation (*n* = 3). Fiber diameter was estimated from at least 10 SEM micrographs.

**Table 2:** Thermal properties of the electrospun fiber mats of 151C and X131A PHBH-based grades in terms of onset degradation temperature (T_5%_), degradation temperature (T_deg_), mass loss at T_deg_, and residual mass at 700 °C.

Sample	T_5%_ (°C)	T_deg_ (°C)	Mass Loss at T_deg_ (%)	Residual Mass at 700 °C (%)
151C	226.9 ± 3.4 ^a^	256.4 ± 10.1 ^a^	65.4 ± 10.2 ^a^	0.42 ± 0.51 ^a^
X131A	230.7 ± 2.1 ^a^	265.4 ± 4.7 ^a^	76.2 ± 5.5 ^a^	0.08 ± 0.12 ^a^

^a^ No significant differences were observed between the samples within the same column (*p* < 0.05). Values are given as mean ± standard deviation (*n* = 3).

**Table 3:** Thermal properties of PHBH-based samples with two different grades (151C and X131A) produced by electrospinning and annealing processes. In the form column, F means fibers and A means annealed.

Grade	Form	First Heating		Cooling		Second Heating			
		Tm (°C)	ΔHm (J/g)	Tc (°C)	ΔHc (J/g)	Tcc (°C)	ΔHcc (J/g)	Tm (°C)	ΔHm (J/g)
151C	F	67.6 ± 12.0 ^a^ (Tm1) 105.9 ± 0.6 ^c^ (Tm2) 125.5 ± 0.1 ^a^ (Tm3) 164.2 ± 0.1 ^a^ (Tm4)	64.4 ± 2.1 ^a^	-	-	65.1 ± 1.6 ^b^	35.4 ± 1.8 ^a^	105.7 ± 1.4 ^c^ (Tm1) 127.2 ± 0.4 ^c^ (Tm2)	44.6 ± 1.6 ^b^
	A	61.4 ± 0.6 ^a^ (Tm1) 108.0 ± 0.5 ^b^ (Tm2) 125.7 ± 0.6 ^a^ (Tm3) 163.2 ± 0.3 ^a^ (Tm4)	61.3 ± 6.8 ^a^	-	-	71.4 ± 1.5 ^a^	34.8 ± 2.8 ^a^	114.5 ± 0.8 ^b^ (Tm1) 133.7 ± 0.4 ^b^ (Tm2) 160.8 ± 1.2 ^a^ (Tm3)	39.9 ± 1.3 ^b^
X131A	F	63.8 ± 0.7 ^a^ (Tm1) 109.6 ± 0.9 ^a,b^ (Tm2) 121.5 ± 0.7 ^b^ (Tm3) 141.8 ± 0.2 ^b^ (Tm4)	69.6 ± 5.7 ^a^	48.3 ± 1.3 ^a^	49.3 ± 8.3 ^a^	51.7 ± 0.3 ^c^	19.8 ± 4.9 ^b^	115.9 ± 1.8 ^b^ (Tm1) 132.1 ± 1.1 ^b^ (Tm2) 141.3 ± 0.6 ^b^ (Tm3)	63.2 ± 3.5 ^a^
	A	66.4 ± 2.1 ^a^ (Tm1) 109.9 ± 0.7 ^a^ (Tm2) 120.4 ± 0.9 ^b^ (Tm3) 140.6 ± 1.1 ^b^ (Tm4)	61.0 ± 3.8 ^a^	52.0 ± 2.9 ^a^	58.7 ± 1.4 ^a^	52.6 ± 0.2 ^c^	10.2 ± 3.5 ^c^	123.1 ± 0.1 ^a^ (Tm1) 144.0 ± 0.0 ^a^ (Tm2)	62.0 ± 1.6 ^a^

^a–c^ Different letters indicate significant differences among the samples within the same parameter (*p* < 0.05). For Tm values, statistical analyses were performed separately for each melting peak. For the remaining thermal parameters, statistical analyses were performed separately within each column. Values are given as mean ± standard deviation (*n* = 3).

**Table 4:** Transparency characteristics of two different grades (151C, X131A) of PHBH-based samples produced via annealing and compression molding.

Grade	Form	*T* (1/mm)	*O* (mm)
151C	Annealed	1.55 ± 0.06 ^b,c^	0.01 ± 0.00 ^c^
	Compression-Molded	1.41 ± 0.17 ^c^	0.03 ± 0.01 ^b^
X131A	Annealed	1.87 ± 0.19 ^b^	0.01 ± 0.00 ^c^
	Compression-Molded	2.73 ± 0.16 ^a^	0.07 ± 0.01 ^a^

^a–c^ Different letters in the same column indicate a significant difference among the samples (*p* < 0.05). Values are given as mean ± standard deviation (*n* = 3).

**Table 5:** Unit-cell parameters (a, b, and c) of the PHB-like lattice, crystallinity, and interplanar distances for PHBH-based films and biopapers of 151C and X131A grades, produced via compression molding and annealing methods.

Grade	Form	PHB-Like Lattice (Å)			Crystallinity (%)	Interplanar Distance (Å)	
		**a**	**b**	**c**	**-**	**d020**	**d110**
151C	Annealed	5.66	12.99	5.83	35	6.50	5.19
	Compression-molded	5.70	13.10	5.92	38	6.55	5.23
X131A	Annealed	5.65	12.98	6.07	41	6.49	5.18
	Compression-molded	5.67	13.03	6.08	46	6.52	5.20

**Table 6:** Mechanical properties of 151C and X131A grades of PHBH fibers produced by electrospinning, and biopapers and films obtained via annealing and compression molding methods, respectively.

Grade	Form	E (MPa)	σ_b_ (MPa)	ɛ_b_ (%)	Toughness (mJ/m^3^)
151C	Fibers	512 ± 96 ^x^	7.8 ± 1.4 ^x^	70.4 ± 15.7 ^x^	5.73 ± 1.05 ^x^
	Annealed	1353 ± 55 ^a^	25.1 ± 1.8 ^b^	14.1 ± 2.4 ^a^	2.03 ± 0.36 ^a^
	Compression-molded	1122 ± 53 ^b^	23.0 ± 0.9 ^b^	12.2 ± 1.2 ^a^	1.53 ± 0.45 ^a,b^
X131A	Fibers	528 ± 157 ^x^	6.3 ± 0.9 ^x^	43.2 ± 7.4 ^y^	2.77 ± 0.34 ^y^
	Annealed	1890 ± 72 ^c^	26.8 ± 1.8 ^a,b^	2.5 ± 0.4 ^b^	0.48 ± 0.14 ^b,c^
	Compression-molded	1692 ± 98 ^d^	30.9 ± 2.2 ^a^	3.9 ± 0.5 ^b^	0.73 ± 0.16 ^c^

^x,y^ For fiber samples, different letters x, y within the same column indicate significant differences between grades (*p* < 0.05). ^a–d^ For film samples (annealed biopapers and compression-molded films), different letters a–d within the same column indicate significant differences among the film groups (*p* < 0.05). Values are given as mean ± standard deviation (*n* = 5). Statistical analyses for fibers and films were performed separately.

**Table 7:** Permeability of PHBH-based films and biopapers of 151C and X131A grades, produced via compression molding and annealing methods.

Grade	Form	WVP × 10^14^ (kg·m·m^−2^·Pa^−1^·s^−1^)	OP × 10^19^ (m^3^.m·m^−2^·Pa^−1^·s^−1^)
151C	Annealed	0.77 ± 0.05 ^a^	14.26 ± 1.04 ^a^
	Compression-molded	0.51 ± 0.02 ^b^	11.52 ± 2.50 ^a,b^
X131A	Annealed	0.58 ± 0.09 ^b^	8.77 ± 0.37 ^b,c^
	Compression-molded	0.33 ± 0.01 ^c^	6.35 ± 0.34 ^c^

^a–c^ Different letters in the same column indicate a significant difference among the samples (*p* < 0.05). Values are given as mean ± standard deviation (*n* = 3).

## Data Availability

The data supporting the findings of this study are available within the article. Additional information can be obtained from the corresponding author upon request.

## Supplementary Materials

The following supporting information can be downloaded at: https://www.mdpi.com/article/10.3390/polym18091061/s1, Figure S1: SEM images of the top view of the compression-molded films, (a) 151C, (b) X131A; Figure S2: Differential scanning calorimetry (DSC) curves, (a) first heating, (b) cooling, and (c) second heating of the compression-molded PHBH films of 151C (top row), and X131A (bottom row); Table S1: Thermal properties of PHBH-based samples with two different grades (151C and X131A) produced by compression molding.
