# Supplementary material for: Development and Characterization of Electrospun Poly(3-hydroxybutyrate-co-3-hydroxyhexanoate) (PHBH) Biopapers
Source: Polymers (Basel). 2026 Apr 28;18(9):1061. doi: 10.3390/polym18091061 (PMC13165099; doi:10.3390/polym18091061)
Supplement: Supplementary file 1 [file polymers-18-01061-s001.zip › polymers-4233283-supplementary.pdf]

Supplementary Materials

Article

Development and Characterization of Electrospun Poly(3-hydroxybutyrate-co-3-hydroxyhexanoate) (PHBH) Biopapers

Ahmet Ozan Basar [1,2,*], Cristina Prieto [1], Luis Cabedo [3], Chris Sammon [4], and Jose Maria Lagaron [1,*]

[1] Novel Materials and Nanotechnology group, Institute of Agrochemistry and Food Technology (IATA), Spanish Council for Scientific Research (CSIC), Paterna 46980, Spain; cprieto@iata.csic.es

[2] PhD in Food Science, Technology and Management, Universitat Politècnica de València, Valencia 46022, Spain

[3] Polymers and Advanced Materials Group (PIMA), School of Technology and Experimental Sciences, Universitat Jaume I (UJI), Castellón 12071, Spain; lcabedo@uji.es

[4] Materials and Engineering Research Institute, Sheffield Hallam University, Sheffield S1 1WB, United Kingdom; c.sammon@shu.ac.uk

[*] Correspondence: aobasar@iata.csic.es (A.O.B.); lagaron@iata.csic.es (J.M.L.)

EXPERIMENTAL RESULTS

Morphological characterization

Morphology of the compression-molded 151C and X131A film was shown in Figure S1 a and b, respectively. At the given thermo-compressing temperature, which was 155 °C for X131A and 175 °C for 151C, both films exhibited a smooth and fracture-free surface.

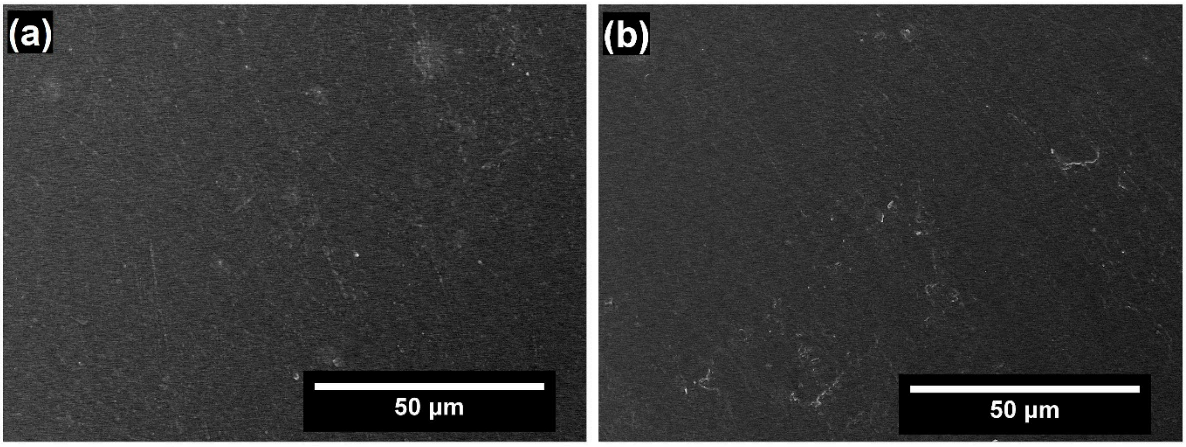

Figure S1. SEM images of the top-view of the compression-molded films, (**a**) 151C, (**b**) X131A.

Thermal characterization by DSC

Figure S2 illustrates the thermal behavior, including melting and crystallization processes, of PHBH-based samples, with the detailed thermal parameters summarized in Table S1. As shown, thermo-compressed films yielded higher melting temperatures and enthalpies compared to fiber and annealed electrospun films (see Table 3 in the main manuscript). This distinction becomes more evident during the subsequent cooling phases, where the thermo-compressed samples demonstrate markedly different thermal characteristics. Specifically, electrospun fibers and films made from 151C and X131A grades show a less pronounced crystallization event than compression-molded films, which display a more intense and sharper crystallization peak. This indicates a more rapid and easier crystallization process in compression-molded films. Furthermore, in the second heating cycles, a notable difference emerges, especially with the X131A-grade samples. Electrospun X131A fibers and films undergo a cold-crystallization event, unlike their compression-molded counterparts, suggesting that the latter were able to achieve complete crystallization during the cooling phase, a capability not observed in the electrospun materials.

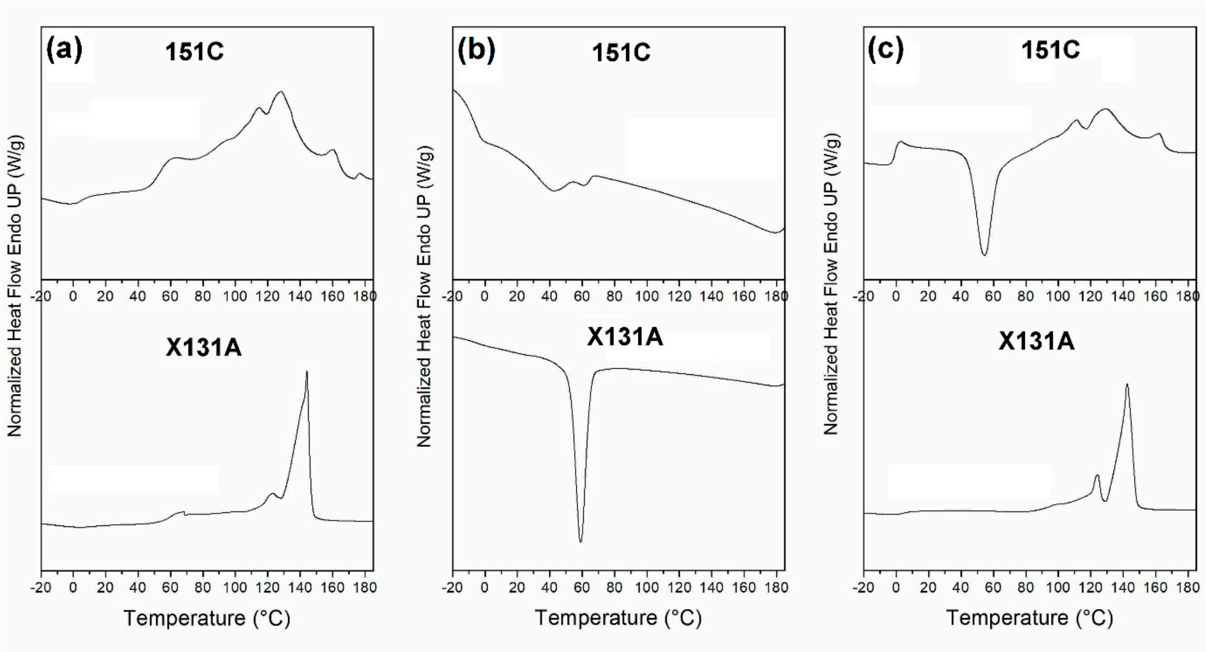

Figure S2. Differential scanning calorimetry (DSC) curves, (**a**) first heating, (**b**) cooling, and (**c**) second heating of the compression-molded PHBH films of 151C (top row), and X131A (bottom row).

Table S1. Thermal properties of PHBH-based samples with two different grades (151C and X131A) produced by compression molding.

Grade	First Heating		Cooling		Second Heating			
	**Tm (°C)**	**ΔHm (J/g)**	**Tc (°C)**	**ΔHc (J/g)**	**Tcc (°C)**	**ΔHcc (J/g)**	**Tm (°C)**	**ΔHm (J/g)**
151C	60.3 ± 0.7 (Tm1)<br>114.2 ± 0.1 (Tm2)<br>128.0 ± 0.1 (Tm3)<br>160.8 ± 0.4 (Tm4)	79.8 ± 0.9	-	-	55.5 ± 0.9	34.1 ± 0.8	111.3 ± 0.2 (Tm1)<br>129.2 ± 0.2 (Tm2)<br>162.6 ± 0.1 (Tm3)	50.1 ± 4.5
X131A	66.4 ± 0.4 (Tm1)<br>122.6 ± 0.7 (Tm2)<br>143.9 ± 0.6 (Tm3)	82.7 ± 0.0	59.1 ± 0.2	72.1 ± 2.8	-	-	124.5 ± 0.1 (Tm1)<br>142.9 ± 0.4 (Tm2)	70.6 ± 2.6
